# Retraction Note: FABP4 contributes to renal interstitial fibrosis via mediating inflammation and lipid metabolism

**DOI:** 10.1038/s41419-021-04084-x

**Published:** 2021-08-20

**Authors:** Yujie Qiao, Liping Liu, Lianhong Yin, Lina Xu, Zeyao Tang, Yan Qi, Zhang Mao, Yanyan Zhao, Xiaodong Ma, Jinyong Peng

**Affiliations:** 1 College of Pharmacy, Dalian Medical University, Western 9 Lvshunnan Road, 116044 Dalian, China; 2 Department of Pharmacy, The First Affiliated Hospital of Dalian Medical University, 116011 Dalian, China; 3 Key Laboratory for Basic and Applied Research on Pharmacodynamic Substances of Traditional Chinese Medicine of Liaoning Province, Dalian Medical University, Dalian, China; 4 National-Local Joint Engineering Research Center for Drug Development (R&D) of Neurodegenerative Diseases, Dalian Medical University, Dalian, China

Retraction Note to: *Cell Death & Disease* 10.1038/s41419-019-1610-5 published online 16 May 2019

The Editors-in-Chief have retracted this article because concerns have been raised regarding some of the figures. An investigation by the College of Pharmacy Dalian Medical University has established that during the course of revision, the bands of ACOX1 and ACADL in rats were repeatedly pasted in Fig. 3C. In the Oil Red O staining result of NFK-49F cells treated with/without LPS + siFABP4 in Fig. 4E, and immunofluorescence result of α-SMA and COL1A expressions in NRK-49F cells treated without LPS + siFABP4 in Fig. 4D, incorrect photos were provided due to file placement confusion. While the institutional investigation has not found evidence of misconduct, the Editors-in-Chief have found that, due to the errors, the findings are no longer reliable. The authors all agree with this retraction.

